# Characterization of β-Glucans from Cereal and Microbial Sources and Their Roles in Feeds for Intestinal Health and Growth of Nursery Pigs

**DOI:** 10.3390/ani13132236

**Published:** 2023-07-07

**Authors:** Hyunjun Choi, Sung Woo Kim

**Affiliations:** Department of Animal Science, North Carolina State University, Raleigh, NC 27695, USA

**Keywords:** β-glucan, growth performance, intestinal health, prebiotics, swine

## Abstract

**Simple Summary:**

The use of antibiotics in animal feeds has been phased out due to concerns surrounding microbial resistance to antibiotics. β-glucans have been shown to improve the intestinal health and growth performance of nursery pigs. β-glucans are non-starch polysaccharides originating from the cell walls of various sources including yeast, bacteria, fungi, and cereal grains. Depending on the sources and dose levels of β-glucans, however, their impacts on intestinal health and growth were not consistent due to the quantitative, compositional, and structural differences of β-glucans. Cereal grains-based diets provide high amounts of soluble fractions of β-glucans, causing digesta viscosity in the GIT of pigs and interfering with the nutrient digestion and intestinal health of pigs. Microbial β-glucans, however, showed positive effects on the intestinal health and growth of nursery pigs. Microbial β-glucans affect the intestinal immune system through activating dectin-1 and toll-like receptors related to the intestinal health of nursery pigs. Therefore, this review investigated the quantitative, compositional, and structural differences of β-glucans and the functional roles of β-glucans in the intestinal health and growth efficiency of nursery pigs.

**Abstract:**

The objectives of this review are to investigate the quantitative, compositional, and structural differences of β-glucans and the functional effects of β-glucans on the intestinal health and growth of nursery pigs. Banning antibiotic feed supplementation increased the research demand for antibiotic alternatives to maintain the intestinal health and growth of nursery pigs. It has been proposed that β-glucans improve the growth efficiency of nursery pigs through positive impacts on their intestinal health. However, based on their structure and source, their impacts can be extensively different. β-glucans are non-starch polysaccharides found in the cell walls of yeast (*Saccharomyces cerevisiae*), bacteria, fungi (*Basidiomycota*), and cereal grains (mainly barley and oats). The total β-glucan content from cereal grains is much greater than that of microbial β-glucans. Cereal β-glucans may interfere with the positive effects of microbial β-glucans on the intestinal health of nursery pigs. Due to their structural differences, cereal β-glucans also cause digesta viscosity, decreasing feed digestion, and decreasing nutrient absorption in the GIT of nursery pigs. Specifically, cereal β-glucans are based on linear glucose molecules linked by β-(1,3)- and β-(1,4)-glycosidic bonds with relatively high water-soluble properties, whereas microbial β-glucans are largely linked with β-(1,3)- and β-(1,6)-glycosidic bonds possessing insoluble properties. From the meta-analysis, the weight gain and feed intake of nursery pigs increased by 7.6% and 5.3%, respectively, through the use of yeast β-glucans (from *Saccharomyces cerevisiae*), and increased by 11.6% and 6.9%, respectively, through the use of bacterial β-glucans (from *Agrobacterium* sp.), whereas the use of cereal β-glucans did not show consistent responses. The optimal use of yeast β-glucans (*Saccharomyces cerevisiae*) was 50 mg/kg in nursery pig diets based on a meta-analysis. Collectively, use of microbial β-glucans can improve the intestinal health of nursery pigs, enhancing immune conditions, whereas the benefits of cereal β-glucans on intestinal health were not consistent.

## 1. Introduction

Weaning is considered the most critical period for nursery pigs, as piglets are exposed to a new environment, are separated from their dam, struggle with new pen mates, and transition from milk to solid feeds, which all negatively affect their overall health, intestinal immune status, and growth performance [1,2]. Antibiotics have been used in nursery feeds to mitigate the negative effects of weaning stress and to improve the intestinal health and growth of nursery pigs. Due to concerns about antibiotic-resistant bacteria, however, the use of antibiotics in feeds has been phased out in many countries [3]. Thus, there is a demand for the investigation of feed additives to reduce the usage of antibiotics and to improve the growth rate of pigs [4]. β-glucans, non-starch polysaccharides (NSP) in cereal grains and microorganisms, have been proposed as a potential means of improving the intestinal health and growth of nursery pigs [5,6]. However, cereal and microbial β-glucans (yeast, bacteria, and other origins) have compositional and structural differences [7].

Cereal β-glucans are based on linear glucose molecules linked by β-(1,3)- and β-(1,4)-glycosidic bonds with relatively high water-soluble properties, whereas yeast β-glucans (from *Saccharomyces cerevisiae*) are largely linked with β-(1,3)- and β-(1,6)-glycosidic bonds possessing insoluble properties [8,9]. Moreover, the total β-glucan content from microbial β-glucans (yeast, bacteria, and algae) is lower compared with the levels found in cereal grain-based diets. Due to these differences, cereal β-glucans can cause increased viscosity of digesta and negatively impact feed digestion in nursery pigs [10], whereas microbial β-glucans may not have those effects. Therefore, the objectives of this review are to investigate the compositional and structural differences between cereal and microbial β-glucans, to provide an overview of the functional effects of microbial β-glucans on intestinal health and growth of nursery pigs, and to investigate the potential application of microbial β-glucans as a feed additive for growth of nursery pigs.

## 2. Difference of Composition and Structure of **β**-Glucans Influence Viscosity of Digesta in GIT of Nursery Pigs

β-glucans are NSP that make up a component of cell walls. β-glucans are derived from yeast (*Saccharomyces cerevisiae*), bacteria, fungi, and cereal grains (mainly from barley and oats) [7]. Those β-glucan sources can cause increased viscosity of digesta in the gastrointestinal tract (GIT) of pigs. Viscosity of digesta in the GIT of nursery pigs, however, can be influenced by the structure, amounts, purity, and molecular weight of β-glucans [9]. Therefore, understanding the compositional and structural differences in β-glucan sources is critical to investigating their effects on the intestinal health and growth of nursery pigs.

### Structural and Compositional Difference of β-Glucans

Barley and oats contain generally higher content of β-glucans than other cereal feedstuffs [11]. The β-glucan content from barley was 5 to 11%, and 3 to 7% from oats [12]. In cereal grains, β-glucans are present in endosperm and sub-aleurone cell walls [7], which require breakdown during the digestion process in pigs.

**Figure 1:**
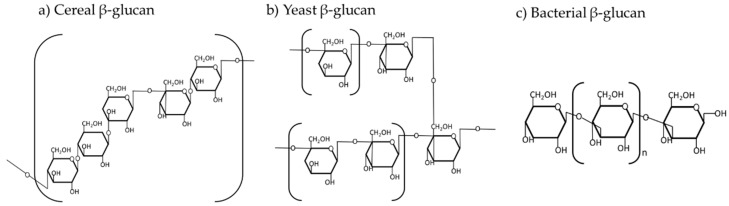
Structural and branching degree of β-glucans from different sources: (**a**) cereal β-glucans (linked with β-(1,3)- and β-(1,4)-glycosidic bonds); (**b**) yeast β-glucans (linked with β-(1,3)- and β-(1,6)-glycosidic bonds); and (**c**) bacterial β-glucans (linked with β-(1,3)-glycosidic bonds). The concept used in this figure was adapted from Du et al. [20].

Cereal β-glucans are based on linear glucose molecules linked with β-(1,3)- and β-(1,4)-glycosidic bonds with relatively high water-soluble properties in the digesta of animals [7] (Figure 1). However, the β-(1,3)- to β-(1,4)-glycosidic bonds ratio of barley is greater than that of oats. In β-glucans, the β-(1,3)-glycosidic bonds are relatively more fermentable than β-(1,4)-glycosidic bonds in the digesta, and the lower molecular weight of β-glucans also increases the fermentation in the digesta of pigs [13]. Barley had a greater proportion of β-(1,3)-glycosidic bonds and a lower molecular weight than oats [14], which may result in higher water-soluble digesta in pigs fed barley-based diets than that in pigs fed oat-based diets [15]. A previous study showed that the β-glucans of barley are already 80% depolymerized in the small intestine of pigs [13]. Moreover, the ileal digestibility of barley β-glucans ranged from 63 to 72%, and the total tract digestibility ranged from 89 to 93%, indicating that most of the β-glucans in barley are digested in the small intestine of pigs [16]. Thus, β-glucans in barley may have greater water solubility in the GIT of pigs compared with that in oats.

Unlike cereal β-glucans, yeast β-glucans (from *Saccharomyces cerevisiae*) are largely linked with β-(1,3)- and β-(1,6)-glycosidic bonds, which contain 53 to 83% of the insoluble fraction [7]. However, structural differences also exist within the microbial β-glucans, which can affect the viscosity in the digesta of nursery pigs. The β-(1,3)-glycosidic bonds are relatively soluble, whereas β-(1,6)-glycosidic bonds are less soluble in the digesta of pigs [17]. Laminarin, a β-glucan derived from algae, is extensively linked with β-(1,3)-glycosidic bonds randomly attached to β-(1,6)-glycosidic bonds, making it relatively soluble and thus causing viscosity in the digesta of pigs. However, laminarin from *Laminaria hyperborean* is interestingly less fermentable due to fewer β-(1,3)-bonds not causing viscous digesta in pigs [18]. The β-glucans from yeast (*Saccharomyces cerevisiae*) mainly consist of branched β-(1,3)-linkage bonds and generally have greater molecular weight compared with Laminarin [19]. The structure of the bacterial β-glucan (from *Agrobacterium* sp.) mainly consists of linear β-(1,3)-glycosidic bonds. Therefore, considering the structural difference among the microbial β-glucans, yeast β-glucans (from *Saccharomyces cerevisiae*) generally have less soluble properties than algal and bacterial β-glucans in the GIT of nursery pigs.

Quantitative contributions of β-glucans in typical feed fed to pigs are mainly from cereal grains (~30 g/kg feed) [21,22,23] rather than microbial feed additives (~1 g/kg feed) (Tables 1–3). Considering the property of β-glucans from microorganisms, the use of microbial feed additives would not cause viscosity issues in the GIT of pigs. Viscosity refers to the ability of mixed fluids (digesta) and soluble polysaccharides such as gums, pectin, and β-glucans to thicken or form gels in the GIT of pigs [24]. In pigs fed diets with highly soluble NSP, the viscosity of digesta was increased [25,26]. Specifically, in pigs fed barley-based diets, the viscosity of digesta in the stomach and ileum was greater when compared with corn-based diets [27]. This is likely due to the high content of soluble NSP in barley [28]. Moreover, barley-based diets also increased the viscosity of digesta in the small intestine of nursery pigs, which can result in a higher incidence of enterotoxigenic *Escherichia coli* (ETEC) infections [29] and reduced feed digestion [30]. Exogenous enzymes can degrade the NSP fractions to reduce viscosity of digesta of nursery pigs [30,31]. However, the viscosity of digesta was not decreased by enzyme supplementation of nursery pigs fed diets containing 50% barley [32]. Additionally, 10% oat-derived β-glucans did not affect the viscosity of digesta, except in the stomach [33]. The possibility of diverse outcomes is likely due to the high depolymerization of the β-glucans from various sources in the GIT of pigs [34]. The depolymerization of cereal β-glucans occurs in the stomach [35], and a high proportion of β-glucans are hydrolyzed in the small intestine of pigs [16,36]. Therefore, some β-glucans in cereal grain-based diet such as processed barley could decrease the intestinal health of nursery pigs by increasing viscosity, whereas microbial β-glucans may not cause increased viscosity and decreased feed digestion.

## 3. Effects of Dietary **β**-Glucans on Intestinal Microbiota and Intestinal Health of Nursery Pigs

The intestinal tract is where feed digestion and absorption occur. Intestinal health is inclusive of seven major criteria: (1) mucosal and luminal microbiota; (2) mucosal inflammation; (3) mucosal oxidative stress; (4) morphological damages and mucosal integrity; (5) crypt stem cell proliferation and tissue repair; (6) effective digestion and absorption of nutrients; and (7) overall well-being and growth efficiency [37]. Among the factors that influence the intestinal health of nursery pigs, feed is highly influential on the intestinal microbiota, intestinal immune responses, and digestion and absorption of nutrients [34,38]. Both cereal and microbial β-glucans (from yeast and bacteria) have been shown to improve the intestinal health of nursery pigs [6,39]. However, some previous studies have not detected the positive effects of dietary β-glucans on intestinal health of nursery pigs, raising questions about the efficacy of β-glucans on the improving intestinal health of pigs. Therefore, this section is focused on the potential of β-glucans to improve the intestinal health of nursery pigs.

### 3.1. Effects of Cereal β-Glucans on Intestinal Health of Nursery Pigs

Supplementation of exogenous β-glucans extracted from cereal grains at 3.5% increased the beneficial microbiota in the ileum, cecum, and colon of pigs [40]. Additionally, barley-derived β-glucans decreased K88-ETEC adhesion to the enterocytes of nursery pigs [41], reducing pathogenic infection in the small intestine. In pigs fed exogenous oat β-glucans, the abundance of *Lactobacillus* spp. and *Bifidobacteria* spp. was greater than in pigs fed exogenous barley β-glucans [40]. Oat β-glucans also increased populations of *Bifidobacteria* spp. and *Lactobacillus* spp. in the stomach and colon of nursery pigs [42]. The reason for different results from β-glucans from cereal grains may be due to the higher insoluble fractions of oats than barley [13]. These studies indicate that cereal β-glucans possess prebiotic effects, modulating the intestinal microbiota and mitigating the negative effects of pathogenic bacterial infection in the GIT of pigs, but the effects of cereal β-glucans could vary. Prebiotics are non-digestible soluble NSP and are fermented by gut microbiota, which potentially enhance the beneficial microbiota in the GIT of pigs [4,43]. However, high levels of soluble β-glucans from barley, especially in processed barley, can cause increased viscosity of digesta and negatively affect microbiota in the GIT of pigs [44]. Moreover, increased viscosity could result in the increased fermentation of pathogenic bacteria related to the post-weaning diarrhea (PWD) of nursery pigs [29,45]. Both barley and oat β-glucan extracts may have beneficial effects on the intestinal microbiota of nursery pigs, but high inclusion rates of high-β-glucan barley in feeds, especially in processed barley, should be used with caution on account of increased digesta viscosity.

### 3.2. Effects of Microbial β-Glucans on Intestinal Health and Growth Performance of Nursery Pigs

Microbial (yeast and bacteria) and algal β-glucans decreased the population of pathogenic bacteria (*Enterobacteria*) in the ileum and colon of pigs [39], indicating the potential role of microbial β-glucans in improving the intestinal health of nursery pigs.

Biological indicators used to determine the inflammation status of the intestine of nursery pigs include decreased levels of pro-inflammatory cytokines (TNF-α, IL-8, IL-6, IL-1β, and IFN-γ) and increased levels of anti-inflammatory cytokines (IL-4, IL-10, and IL-13) [4]. After weaning, the mRNA expression of pro-inflammatory cytokines was increased [46], indicating that weaning stress affects cytokine signaling modulation in the small intestine of nursery pigs [47]. Supplementation of yeast β-glucans reduced pro-inflammatory cytokines and increased anti-inflammatory cytokines in the jejunum of nursery pigs [48]. The potential of microbial β-glucans to improve the immune response may be attributed to the activation of dectin-1 receptor in the intestine through β-(1,3)-glycosidic bonds present in β-glucans [6,49]. The increased dectin-1 receptor stimulation by microbial β-glucans (yeast and bacteria) increased phagocytosis in the immune cells and increased cytokines, modulating the immune response through humoral immunity in pigs [50]. As a result, microbial β-glucans reduce the energy cost of the immune response through the activation of dectin-1 receptor, decrease inflammation in the GIT, and improve the growth rate of nursery pigs [51,52]. Therefore, supplementation of microbial β-glucans could reduce enteric inflammation in the GIT and improve the growth rate of nursery pigs [34].

The effects of microbial β-glucans include (1) reduced pathogenic microbiota in the GIT; (2) increased immune responses (pro-inflammatory cytokines); (3) increased mucosa protein and tight junction protein of enterocytes; and (4) improved morphology of nursery pigs. The possibility for these effects is mainly due to the prevention of enterocyte inflammation in nursery pigs, which increases growth performance [6,10,53]. However, the optimal use of β-glucans may be variable depending on β-glucan sources due to differences in the purity, molecular weight, conformation, chemical structure, and solubility of β-glucans in nursery diets [6]. Therefore, this section investigates the effects of microbial β-glucans on the intestinal health and growth of nursery pigs.

#### 3.2.1. Yeast (*Saccharomyces cerevisiae*)

**Figure 2:**
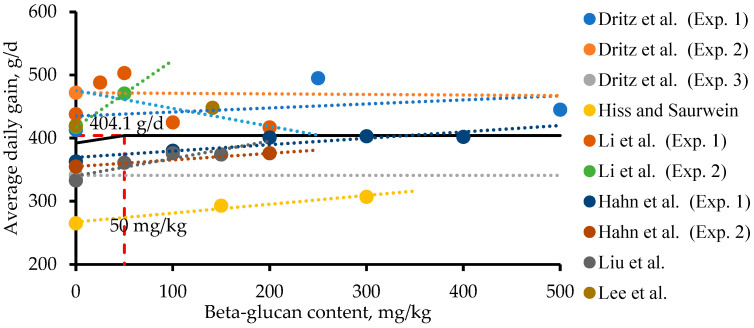
Improvement in body weight gain of nursery pigs fed diets with increasing quantities of yeast (*Saccharomyces cerevisiae*) β-glucans (0 to 1000 mg/kg) using a linear broken line analysis. The meta-analysis is conducted by Proc NLMIXED to determine the breakpoint on the regression of body-weight gain in nursery pigs based on the data from six published studies (ten experiments with a non-challenged period). The breakpoint (a one-slope broken line analysis) was 50 mg/kg (standard error = 0.561; *p* < 0.05) of β-glucan content in nursery pig diets. The equation for body weight gain in nursery pigs was ADG, g/d = 404.1 − 0.235 × z1 (β-glucan content, mg/kg), R^2^ = 0.87 if β-glucan content is ≥breakpoint, then z1 = 0. Due to the lack of published data for other microbial β-glucans, a meta-analysis was not conducted [10,55,58,61,62].

The use of yeast β-glucans (from *Saccharomyces cerevisiae*) has positive effects on the intestinal health and growth performance of nursery pigs, with an increase in weight gain of 7.6% and an increase in feed intake of 5.3% (Table 1). Yeast β-glucans decreased Enterobacteria spp. in the ileum and proximal colon [39]. Additionally, yeast β-glucans improved the morphology parameters of nursery pigs such as VH:CD and jejunum goblet cells [54] and increased the digestibility of nutrients for nursery pigs [55]. The reason for the improvement in the intestinal health of nursery pigs is likely due to the activation of the dectin-1 receptor in the small intestine. However, yeast β-glucans (from *Saccharomyces cerevisiae*) did not linearly improve the growth performance of nursery pigs with increasing β-glucan levels [10,53]. The reason for the growth of pigs showing quadratic changes through yeast β-glucans (from *Saccharomyces cerevisiae*) could be due to high immune stimulation increasing energy use for body maintenance [52,56,57]. During the period of high immune stimulation, proinflammatory cytokines such as TNF-α, IL-6, and IL-1 are released to activate macrophages for defense against infection in pigs [10,58,59]. The supplementation of yeast β-glucans (from *Saccharomyces cerevisiae*) showed quadratic responses in the growth performance, IL-1, and TNF-α in broiler chickens [57]. In the case of an in vitro study using macrophages from mice, zymosan (a form of yeast β-glucan) increased TNF-α secretion [60]. The optimal use of yeast β-glucans (from *Saccharomyces cerevisiae*) could be considered to improve the immune responses of nursery pigs related to growth performance. In this review, the optimal use of yeast β-glucans (from *Saccharomyces cerevisiae*) was determined as 50 mg/kg of nursery diets (Figure 2). In summary, yeast β-glucans (from *Saccharomyces cerevisiae*) have the potential to increase the intestinal health and growth performance of nursery pigs, showing decreased pathogenic bacteria in the GIT, improved morphology parameters, and increased nutrient digestibility.

A meta-analysis was conducted to determine the optimal use of yeast β-glucans (from *Saccharomyces cerevisiae*) in feeds based on the growth performance data of nursery pigs. A total of 29 datasets with body weight (BW), average daily gain (ADG), average daily feed intake (ADFI), and gain to feed ratio (G:F) from six published research papers with ten experiments were used. For the literature search in PubMed, Web of Science, and Google Scholar, the used keywords were β-glucans, growth performance, intestinal health, and nursery pigs. The found papers were manually screened based on the title and experimental procedures. During this screening process, data from growing pigs or sows were excluded. Additionally, papers which did not contain information about specific levels of β-glucans in the test product were not included in the meta-analysis. For the meta-analysis, the inclusion rate of yeast β-glucans (from *Saccharomyces cerevisiae*) with respect to the growth response was evaluated with a one-slope broken line analysis using the Proc NLMIXED procedure in SAS (SAS Inst. Inc., Cary, NC, USA) [63]. Using a one-slope broken line analysis, the optimal use of yeast β-glucans in feeds for the ADG of nursery pigs was obtained. Statistical significance and tendency were declared at *p* < 0.05 and 0.05 ≤ *p* < 0.10, respectively. The optimal use of yeast (*Saccharomyces cerevisiae*) β-glucans in nursery pig diets was 50 mg/kg (Figure 2). For other microbial β-glucans, a meta-analysis of their optimal use was not conducted due to the limited amount of data.

**Table 1:** Effects of the use of yeast β-glucans (from *Saccharomyces cerevisiae*) on the intestinal health and growth performance of nursery pigs ^1,2^.

**Item**	**Initial BW (kg) or Age (d)**	**Experimental Period (d)**	**β-Glucan Compound (mg/kg)**	**β-Glucan (mg/kg)**	**Results**			**Reference**
Intestinal health	8.0 kg	28	500	141	Increased jejunal goblet cells, tended to decrease diarrhea during d 0 to 14, tended to increase VH:CD, and tended to increase apparent ileal and total tract digestibility of energy			[54]
	6.4 kg	35	-	100, 200, 300, and 400	Linearly increased apparent total tract digestibility of nutrients			[55]
	5.8 kg	21	-	50, 100, and 150	Increased villus height and VH:CD on the jejunum			[61]
	15.3 kg	28	-	250	Decreased *Enterobacteria* spp. In ileum and proximal colon			[39]
**Item**	**Initial BW (kg) or age (d)**	**Experimental period (d)**	**β-glucan compound (mg/kg)**	**β-glucan (mg/kg)**	**ADG (% change)**	**ADFI (% change)**	**G:F (% change)**	**Reference**
Growth performance	4.9 kg	28	-	250	19.9 **	23.2 **	−1.1	[58]
				500	7.7	11.6	−1.1	
	5.0 kg	28	-	1000	−1.9	−7.1 **	2.6	
				1000	0	−1.5 **	0	
	28 d	28	-	150	10.6	7.4	0	[62]
				300	15.8	15.4 *	0	
	8.7 ^3^ kg	28	-	25	11.4	7.5	3.1	[10]
				50	14.8	11.6	2.7	
				100	−3	−4.6	0.6	
				200	−4.8	−3.7	−1.3	
	8.2 kg	28	-	50	12.7 **	11.5 **	1.3	
	6.4 ^4^ kg	35	-	100	4.7	3.2	1.6	[55]
				200	10.5	9	0	
				300	11	6.8	3.2	
				400	10.7	5.4	4.8	
	6.2 kg	35	-	200	5.9	1.8	4.2	
	5.8 kg	21	-	50	8.4 **	2.4	5.9	[61]
				100	12.9 **	6.0 **	6.6	
				150	12.3 **	8.8	3.2	
	8.0 kg	28	500	141	6.7 *	2.8	3.8	[54]
	6.0 kg	35	2000	NA	7.4 **	6.5 **	0.9	[64]
	6.0 kg	48	2000	NA	−5.5	−6.9	1.6	[65]
	Average % change:				7.6	5.3	1.9	

BW, body weight; NA, not available; VH:CD, villus height to crypt depth ratio. ^1^ Asterisk marks (*, **) represent statistical tendency (*p* < 0.10) and significant difference (*p* < 0.05), respectively. ^2^ The percentage increase or decrease in the average daily gain (ADG), average daily feed intake (ADFI), and gain-to-feed ratio (G:F) was determined in beta-glucan supplementation groups relative to the control group. ^3^ β-glucan supplementation contents quadratically increased (*p* < 0.05) the ADG of nursery pigs. ^4^ β-glucan supplementation contents linearly tended to increase (*p* < 0.10) the ADG of nursery pigs.

#### 3.2.2. Bacteria (*Agrobacterium* sp.)

The supplementation of bacterial β-glucans (from *Agrobacterium* sp.) showed positive effects on the intestinal health and growth performance of nursery pigs, resulting in an 11.6% increase in weight gain and a 6.9% increase in the feed intake of nursery pigs (Table 2). Specifically, the supplementation of 50 mg/kg bacterial β-glucans (from *Agrobacterium* sp. *ZX09*) in feeds increased villus height, decreased crypt depth, and increased VH:CD after lipopolysaccharide (LPS) challenge [6]. Moreover, the 50 mg/kg of bacterial β-glucans (from *Agrobacterium* sp. *ZX09*) decreased the intestinal permeability of the small intestine of nursery pigs [61]. The intestinal permeability function can be determined by tight junction proteins such as occludin, claudin, and MUC1 and 2. High tight junction protein complexes between intestinal cells inhibit the paracellular flow, thus enhancing pathogen prevention [4]. Additionally, the highly viscous mucus in the intestine, consisting of cross-linked mucins, antimicrobial factors, and trefoil peptides, acts as an additional physical and chemical intestinal barrier and prevents microorganisms from making contact with the intestinal epithelium [48]. The reason for the decrease in intestinal permeability is likely the activation of dectin-1 receptor. The increase in dectin-1 receptor in the intestine can increase phagocytosis in immune cells and cytokine production, which can improve the intestinal health of nursery pigs. Lastly, bacterial β-glucans (from *Agrobacterium* sp.) linearly increased IL-10 and linearly decreased TNF-α in the jejunum mucosa of nursery pigs. As prebiotics effects of β-glucans in the intestinal microbiota of pigs, supplementation with 200 mg/kg of bacterial β-glucans (from *Agrobacterium* sp.) increased the relative abundance of *Fournierella*, *Parabacteria*, and *Alistipes* in the ileum, providing growth substrates with alpha-glucosidase activity, and increased *Oscillospira*, a butyrate-producing bacteria [66]. Additionally, supplementation with 300 mg/kg of bacterial β-glucans (from *Agrobacterium* sp.) showed interaction with morphological parameters (villus height), the expression genes related to intestinal integrity (Z0-1, Occludin-1, and MUC2), and the growth performance of nursery pigs challenged with ETEC [67], indicating that bacterial β-glucans can be highly effective under challenged conditions in mitigating pathogenic bacteria infection [48]. In terms of the growth of nursery pigs, bacterial β-glucans (from *Agrobacterium* sp.) also showed a quadratic response (as was shown in the yeast β-glucans (from *Saccharomyces cerevisiae*)) [6], which indicates that bacterial β-glucans also require optimal usage in order to improve intestinal health and growth. However, due to the lack of published data, the optimal use of bacterial β-glucans cannot be determined. In summary, bacterial β-glucans can decrease intestinal permeability, which can prevent pathogenic bacteria infections and improve the intestinal health and growth performance of nursery pigs.

#### 3.2.3. Algae (*Euglena gracilis*, *Laminaria digitata*, and *Laminaria hyperborea*)

The use of algal β-glucans has been shown to improve the intestinal health of nursery pigs by decreasing intestinal permeability in jejunal mucosa and decreasing pathogenic bacteria such as *Enterobacteria* spp. (Table 3), but it did not improve growth performance [54]. Specifically, 54 mg/kg of algal β-glucans increased mRNA abundance, representing a decrease in intestinal permeability (claudin, occludin, and MUC2) in the jejunal mucosa of nursery pigs [49]. Additionally, 108 mg/kg of algal β-glucans increased the mRNA abundance of dectin-1 receptors in the jejunal mucosa, and 141 mg/kg of β-glucans also increased the relative gene expression of tight junction proteins (claudin, occludin, and MUC1) in the jejunum of nursery pigs. Lastly, microbiota data showed that 250 mg/kg of 2 algal β-glucans (from *Laminaria digitata* and *Laminaria hyperborea*) decreased *Enterobacteria* spp. In the ileum and proximal colon of nursery pigs. Several studies showed improvements in the growth performance and intestinal health in pigs fed seaweed extract-supplemented diets (from *Laminaria* spp.) [70,71,72]. However, information on the algal β-glucans content in the seaweed extract was not available. Further research is needed to investigate the effects of algal β-glucans on the growth performance of nursery pigs.

## 4. Conclusions

Due to their quantitative, compositional, and structural differences, cereal β-glucans have relatively high water-soluble properties, whereas microbial β-glucans (yeast and bacteria) have water-insoluble properties in the digesta of nursery pigs. The high water-soluble properties of cereal β-glucans, if fed in ample amounts, are shown to cause digesta viscosity, negatively affecting the intestinal health and nutrient utilization in nursery pigs. In contrast, the use of microbial β-glucans showed positive effects on the intestinal health of nursery pigs at an optimal level through mainly activating the dectin-1 receptor and prebiotic effects without causing digesta viscosity. From this review, it is evident that the use of microbial β-glucans can improve intestinal health and nutrient utilization, which, in turn, can improve the growth efficiency of nursery pigs.

## Figures and Tables

**Table 2:** Effects of the use of bacterial β-glucans (from *Agrobacterium* sp. and *Paenibacillus polymyxa*) on the intestinal health and growth performance of nursery pigs ^1,2^.

**Item**	**Initial BW (kg) or Age (d)**	**Experimental Period (d)**	**β-Glucan Compound (mg/kg)**	**β-Glucan (mg/kg)**	**Results**			**Reference**
Intestinal health Bacteria (*Agrobacterium* sp.)	21 d	28	-	50	Increased villus height, decreased crypt depth, and increased VH:CD after LPS challenge; increased mRNA abundance representing intestinal permeability (Z0-1, occludin, claudin, and MUC1 and 2), and decreased malondialdehyde in the jejunal mucosa after LPS challenge			[48]
	7.0 kg	28	-	50, 100, and 200	Linearly increased IL-10 and linearly decreased TNF-α level of jejunal mucosa			[6]
				100	Increased MUC1 and 2 to β-actin mRNA ratio			[6]
	6.1 kg	21	-	200	Increased VH:CD in jejunum and increased mRNA abundance of an intestinal permeability parameter (occludin)			[66]
	6.1 kg	21	500	300	Increased VH:CD in jejunum, increased mRNA abundance of intestinal permeability parameter in jejunum (Z0-1, claudin-1, and MUC2), and increased *Lactobacillus* spp. and propionic acid in cecum digesta after ETEC challenge			[67]
					Decreased malondialdehyde, TNF-α, and IL-6 in jejunum after ETEC challenge			[68]
**Item**	**Initial BW (kg) or age (d)**	**Experimental period (d)**	**β-glucan compound (mg/kg)**	**β-glucan (mg/kg)**	**ADG (% change)**	**ADFI (% change)**	**G:F (% change)**	**Reference**
Growth performance	21 d	21	-	50	21.6 **	11.0 **	9.2	[48]
Bacteria (*Agrobacterium* sp.)				50	14.1	8.2	6.6	
	7.0 ^3^ kg	28	-	25	2.5	2.8	−0.6	[6]
				50	10.4	8.0	2.4	[6]
				100	15.7	10.2	4.9	
				200	−0.9	1.0	−2.3	
	6.1 kg	21	-	200	17.6	6.9	4.3	[66]
	Average % change				11.6	6.9	3.5	
Bacteria (*Paenibacillus polymyxa*)	5.6 kg	28		400	5.8 *	−0.8	6.6	[69]

BW, body weight; NA, not available; MUC, mucin; LPS, lipopolysaccharide; VH:CD, villus height to crypt depth ratio; IL-10, interleukin-10; TNF-α, tumor necrosis factor-α; Z0-1, zonula occludens-1. ^1^ Asterisk marks (*, **) represent statistical tendency (*p* < 0.10) and significant difference (*p* < 0.05), respectively. ^2^ The percentage increase or decrease in the average daily gain (ADG), average daily feed intake (ADFI), and gain to feed ratio (G:F) is determined in beta-glucan supplementation groups relative to the control group. ^3^ β-glucan supplementation contents linearly (*p* < 0.05) and quadratically (*p* < 0.05) increased the ADG of nursery pigs.

**Table 3:** Effects of the use of algal β-glucans (from *Euglena gracilis*, *Laminaria digitata*, and *Laminaria hyperborea*) on the intestinal health of nursery pigs.

Item	Initial BW (kg) or Age (d)	Experimental Period (d)	β-Glucan (mg/kg)	Results	Reference
Algae (*Euglena gracilis*)	7.7 kg	17	54	Increased mRNA abundance representing intestinal permeability (claudin, occludin, and MUC2) in jejunal mucosa on d 12	[65]
			108	Increased mRNA abundance representing intestinal permeability (dectin) in jejunal mucosa on d 5 and 12. Decreased transcellular permeability.	
Algae (*Laminaria digitata*)	15.3 kg	28	250	Decreased *Enterobacteria* spp. in ileum and proximal colon; increased acetic acid and decreased propionic acid in ileum	[39]
Algae (*Laminaria hyperborea*)	15.3 kg	28	250	Decreased *Enterobacteria* spp. in ileum and proximal colon; decreased total volatile fatty acid in the ileum	

MUC, mucin; mRNA, messenger ribonucleic acid.

## Data Availability

Not applicable.
